# Post-collisional mantle delamination in the Dinarides implied from staircases of Oligo-Miocene uplifted marine terraces

**DOI:** 10.1038/s41598-021-81561-5

**Published:** 2021-01-29

**Authors:** Philipp Balling, Christoph Grützner, Bruno Tomljenović, Wim Spakman, Kamil Ustaszewski

**Affiliations:** 1 Institute for Geological Sciences, Friedrich-Schiller-University Jena, Burgweg 11, 07749 Jena, Germany; 2 Faculty of Mining, Geology and Petroleum Engineering, University of Zagreb, Pierottijeva 6, 10000 Zagreb, Croatia; 3 Faculty of Geosciences, Utrecht University, Vening Meineszgebouw A Princetonlaan 8a, 3584 CB Utrecht, The Netherlands

**Keywords:** Geodynamics, Geology, Geomorphology, Tectonics

## Abstract

The Dinarides fold-thrust belt on the Balkan Peninsula resulted from convergence between the Adriatic and Eurasian plates since Mid-Jurassic times. Under the Dinarides, S-wave receiver functions, P-wave tomographic models, and shear-wave splitting data show anomalously thin lithosphere overlying a short down-flexed slab geometry. This geometry suggests a delamination of Adriatic lithosphere. Here, we link the evolution of this continental convergence system to hitherto unreported sets of extensively uplifted Oligocene–Miocene (28–17 Ma) marine terraces preserved at elevations of up to 600 m along the Dinaric coastal range. River incision on either side of the Mediterranean-Black Sea drainage divide is comparable to the amounts of terrace uplift. The preservation of the uplifted terraces implies that the most External Dinarides did not experience substantial deformation other than surface uplift in the Neogene. These observations and the contemporaneous emplacement of igneous rocks (33–22 Ma) in the internal Dinarides suggest that the Oligo-Miocene orogen-wide uplift was driven by post-break-off delamination of the Adriatic lithospheric mantle, this was followed by isostatic readjustment of the remaining crust. Our study details how lithospheric delamination exerts an important control on crustal deformation and that its crustal signature and geomorphic imprint can be preserved for millions of years.

## Introduction

The influence of deep-seated processes on deformation patterns and rates in collisional orogens is unequivocally accepted, yet challenging to quantify. It is well established that an interplay between plate convergence and subduction velocity causes subducted slabs to either advance or retreat, exerting first-order control on the orogenic style^1–3^. It is less well understood how the removal of the lithospheric mantle of an orogen modifies that interplay^4^. The mechanism of lithosphere root removal can be related to: (i) sudden or gradual slab break-off or detachment^5,6^; (ii) gradual viscous drip-type lithospheric instability^7^or (iii) thermal attenuation of the lithosphere by asthenospheric upwelling leading to delamination of the lithospheric mantle^8–11^. Delamination is the process that decouples negatively-buoyant lithospheric mantle from buoyant crust, allowing replacement with less dense asthenosphere and leading to surface and Moho uplift and cogenetic magmatism^8^. This can be either achieved by syn-collisional^9^ or by post-collisional delamination^8^. Evidence for delamination is usually only available from geophysical imaging, from geochemical/geochronological data, or from the topographic signal of the crustal response. In this paper we show that the present-day topography of the Dinarides still holds a record of delamination that occurred during the Oligo-Miocene.

### The Dinarides fold and thrust belt

**Figure 1:**
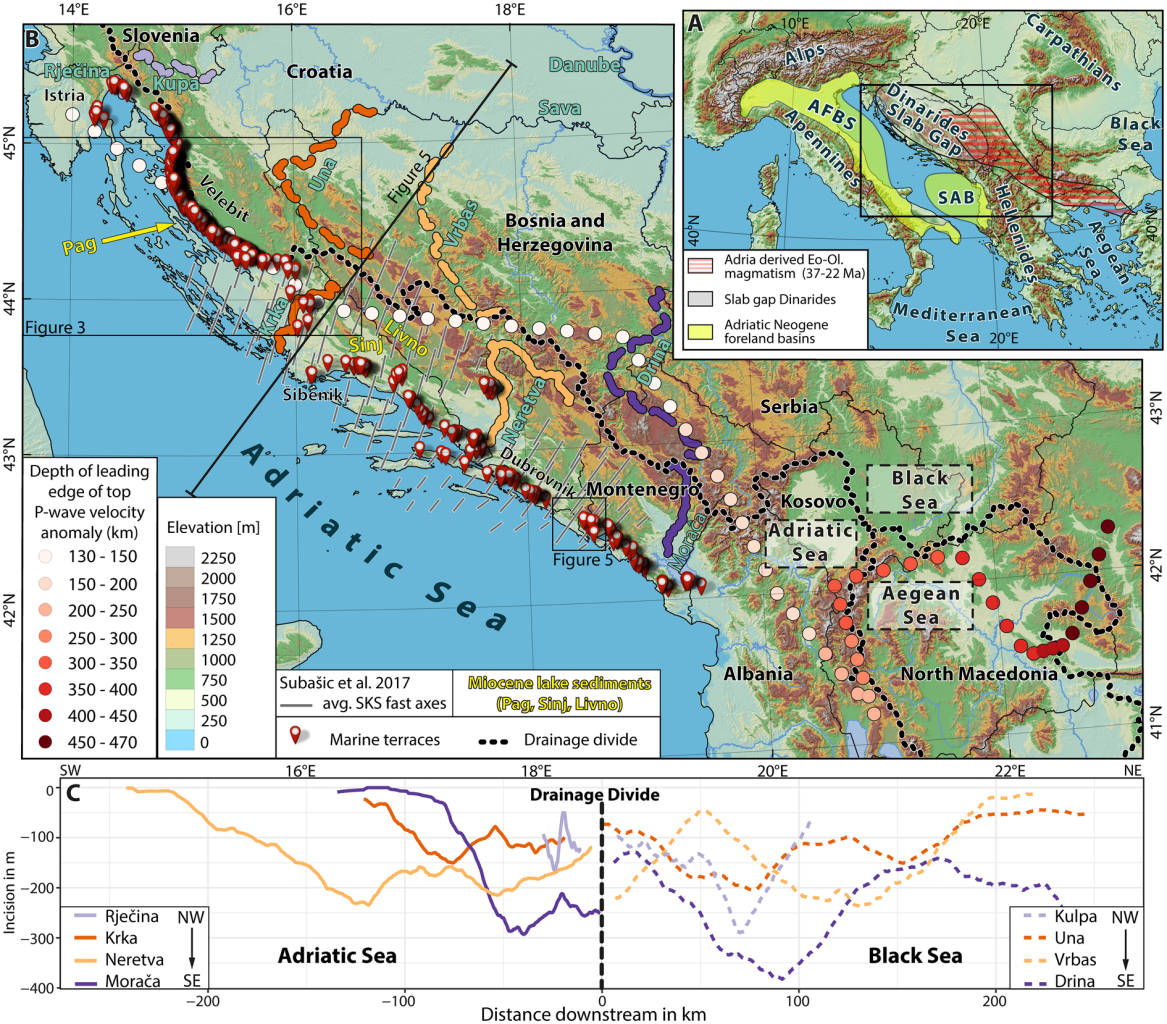
** (a)** DEM^34^ of Circum-Adriatic orogens, location of the Eo-Oligocene magmatic domain^20^ and locations of Neogene foreland basins (AFBS: Apennine Foreland Basin System)^68^; SAB: South Adriatic Basin^54^. **(b)** DEM^34^ with the position of all mapped marine terraces located within the Dinaric slab gap. Spaced dots show the leading edge of the slab top extracted from the mapped positive velocity anomalies of tomography model UU-P07^24,25^. Shear wave splitting axes indicate the direction of orogen-perpendicular mantle flow^26^. Black dotted line shows drainage divide separating the Adriatic, Black and Aegean Sea catchments. Yellow labels point to locations of coastal-near Miocene freshwater sediments. **(c)** Swath profiles along the Dinaric rivers show a symmetric incision across the drainage divide.

**Figure 2:**
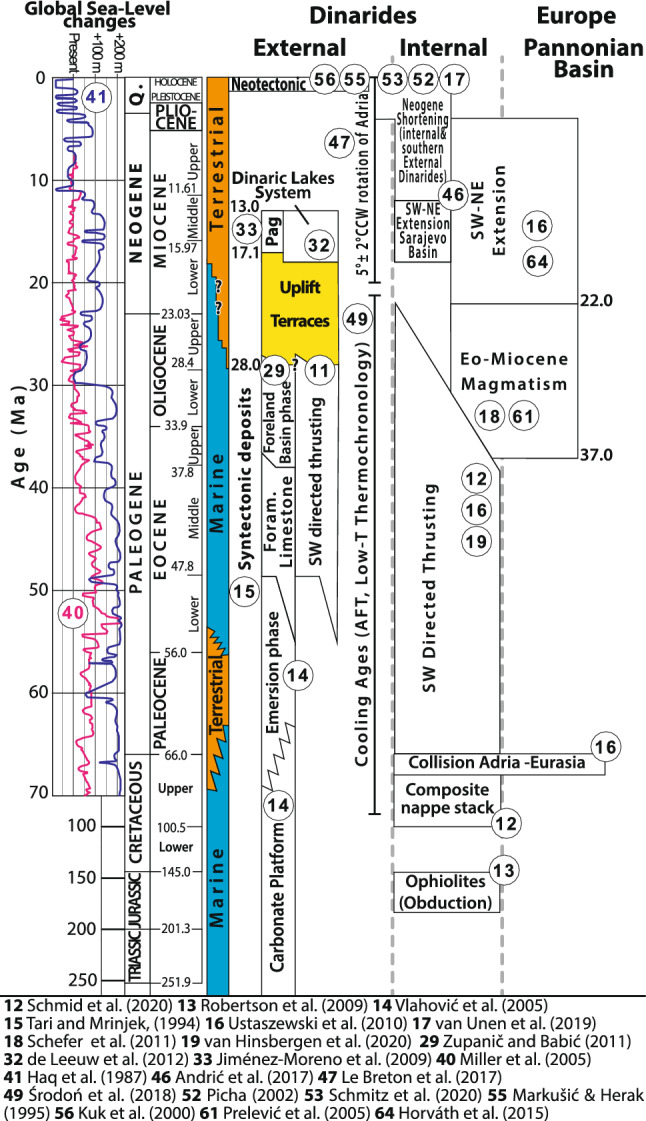
Timetable illustrating major tectonic events in the External and Internal Dinarides and the northerly adjacent Pannonian Basin. The colored chart shows the depositional environment for the External Dinarides. The time span of the formation and uplift of the marine terraces is marked in yellow.

The Dinarides form a SW-directed nappe stack that resulted from convergence between the Adriatic and Eurasian plates since Mid-Jurassic times^12^. They are subdivided into the ophiolite-bearing^13^ Internal and the External Dinarides, the latter mainly built up by Mesozoic platform carbonates^14^ and mid-Eocene–Early Oligocene syn-tectonic sediments^15^. Following Cretaceous oceanic subduction and late Cretaceous continent–continent collision, the Internal Dinarides were the first to undergo crustal shortening during the Paleocene^16^. Propositions for detachment of the oceanic slab vary between the early Oligocene^17^ to late Eocene^18,19^. Slab detachment possibly progressed from the NW to the SE^20^. A short slab reaching depths between 150 and 180 km and the gap left by slab detachment are observed in various P-wave tomography models^21–25^ and in shear-wave splitting (SKS) data^26^. Due to the uniform orogen-perpendicular orientation of the SKS values, the mantle flow at a depth of > 150 km underneath the Dinarides is not governed by the presence of an orogen-parallel deep slab (Fig. 1b, 3D view supplement A1). Such a barrier on the lithospheric scale would rather favor orogen-parallel over orogen- perpendicular mantle flow patterns. Continued shortening, crustal thickening, and foreland flexure in the External Dinarides led to the deposition of Eocene–Oligocene syntectonic deposits^27–31^, subdivided into the proximal coarse-grained molasse (Promina Beds)^27–30^ and the fine-grained distal “flysch” deposits^31^. The most important geological processes shaping the Dinarides are summarized in Fig. 2.

In this study we identify large, flat surfaces on Paleogene proximal syn-tectonic marine deposits and on older Mesozoic Adriatic carbonate platform bedrock along the Dinaric coast (Fig. 1). Such surfaces are absent within Miocene lacustrine sediments that were deposited around 18–13 Ma in a system of intramontane Dinaric lakes formed on top of previously deformed bedrock^32,33^. So far, no geodynamic scenario has explained the occurrence of these conspicuous surfaces in the External Dinarides.

## Methods

In order to map the horizontal surfaces across the External Dinarides, we used the EU-DEM v1.1^34^ to produce slope and roughness maps. Based on these maps and additional topographic profiles, we manually mapped flat surfaces within the range of 0°–8° slope and a roughness of 0–22 m. In a second step only flat surfaces with a lower median slope of not more than 6° and a lower median roughness of not more than 7 m were considered for the final selection. Field mapping was performed to ground-proof these results and to look for major Neogene-Quaternary faults. We then analyzed 1 km-wide swath profiles of four pairs of rivers on both sides of the Dinaric drainage divide to quantify river incision as a proxy for regional uplift using the TopoToolbox2^35^. The 3D P-wave velocity model UU-P07^24,36^(Supplementary Videos A2, A3) served to map the top and the leading edge of the subducted Adriatic lithosphere.

## Results

### Marine terraces and river incision in the external Dinarides

**Figure 3:**
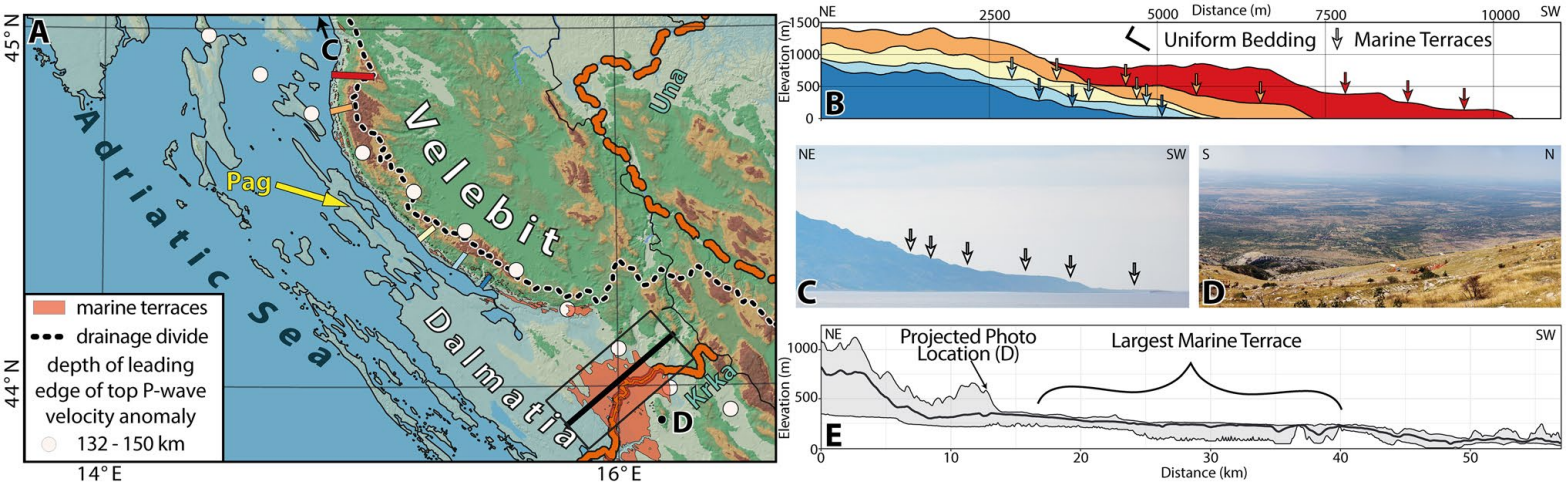
**(a)** DEM^34^ of the Mt. Velebit region, color-coded as in Fig. 1b. Colored lines are section traces along the Mt. Velebit. Black rectangle marks the area of the swath profile across the largest marine terraces (red in Fig. 1b). **(b)** Topographic profiles show staircase morphology. Terraces are marked by arrows. **(c)** Field photo of the staircase morphology of the Velebit Mt. **(d)** Field photo of the largest marine terrace around the Krka River. **(e)** Swath profile showing lateral extent of the largest preserved marine terrace.

**Figure 4:**
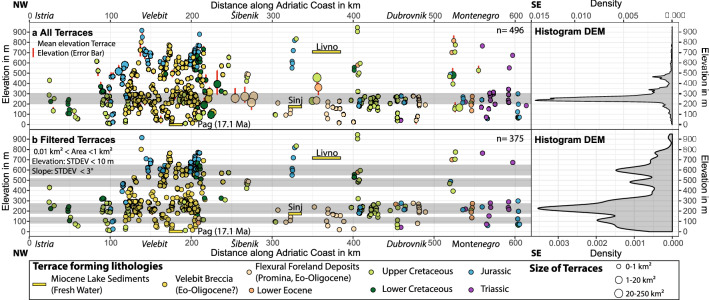
Location and size of terraces versus mean elevation colored according to corresponding lithostratigraphic units. Position and present-day elevation of the Miocene basins are shown; for exact location see Fig. 1b. **(a)** Dataset of all extracted terraces with corresponding histogram of the terrace elevation. **(b)** Filtered dataset showing only terraces > 0.01 km^2^ and < 1 km^2^, and those that have an elevation standard deviation of less than 10 m and a slope standard deviation of less than 3°.

All mapped horizontal surfaces follow the Adriatic shoreline for ca. 600 km from Istria to Albania (Fig. 1, Supplementary KML File, A4). They form a staircase morphology in the Velebit Mountains (Fig. 3), affect different bedrock lithologies, and encompass present-day elevations between 10 and 920 m (Fig. 4). Their morphology is neither influenced by bedding dip nor by faults. The largest surface was identified around the Krka River in the central part of the External Dinarides (Fig. 3). Unfortunately, neither terrestrial nor marine deposits were found on top of these flat surfaces that could irrefutably prove their erosive marine origin. Due to the lack of other denudation processes that would result in large surfaces in close proximity to the present-day shoreline, and which cut folds, faults, and tilted strata on various elevations, we interpret these flat surfaces as degradational, marine wave-cut terraces (Figs. 1, 3). Such marine terraces and the associated staircase morphology are documented worldwide as a result of rather constant uplift and oscillating sea-level variations: thus, they are widely used to reconstruct surface uplift^37–39^(Fig. 1).

The mean present-day elevations of the mapped marine terraces are between 200 and 300 m (Fig. 4A). The histogram of all terrace elevations may be biased by the largest terraces, because multiple measurements for larger terraces were included. Therefore, all terraces with areas smaller than 0.01 km^2^ and larger than 1 km^2^ were excluded from the filtered histogram, as well as those with standard deviations of more than 3° for slope and more than 10 m for elevation. The histogram of the filtered dataset shows four statistically significant peaks for smaller terraces at elevation of 100, 200–300, 450, and 600 m (Fig. 4b). The uneven preservation of marine terraces along the Adriatic coast is related to the different bedrock lithologies and coastal-near high elevations. In the Velebit Mountains, most of the terraces are located within the Velebit Breccia^27^ (Figs. 3, 4). This well cemented and massive carbonate breccia is mainly exposed at the southwestern slope of the Velebit Mountains and covers the mountain tops. This shows that the Velebit Breccia has a higher erosional resistance compared to the surrounding carbonate bedrock. Consequently, this favors the pristine terrace preservation within the Velebit Beccia all across the frontal Velebit Mt. region.

The youngest proximal flexural foreland basin deposits of the External Dinarides are the Promina Beds (Figs. 2, 4). These mid-Eocene (locally early Eocene^30^) to early Oligocene flexural foreland basin sediments were deposited in deep to shallow marine environments that changed to deltaic, lacustrine, and alluvial environments during the Oligocene^29^. Since then, the global sea level was at maximum between 50 m^40^ and 100 m^41^ above the present-day level (Fig. 2). Therefore, the formation of at least all terraces presently preserved at elevations higher than 100 m above sea level must be due to a regional surface uplift of the Dinarides since the Oligocene.

All terraces formed within carbonates. Due to the lack of suitable exposure dating techniques for carbonate rocks, the absolute age dating of terrace formation is nearly impossible. However, not a single terrace was found within the lacustrine Miocene sediments of the Dinaride Lake System. These sediments record the minimum age of the change from marine to terrestrial (lacustrine) conditions^32^ (Fig. 2). Outcrops of Miocene lake sediments on Pag Island (Fig. 1b) are situated at present-day sea level, that is, topographically below all mapped terraces. There is no structural offset between the Miocene strata on Pag Island and the nearby marine terraces of the frontal Velebit Mt. region. Consequently, the marine terraces must be older than the Miocene sediments on Pag Island. The start of Miocene sedimentation on Pag Island is dated by magnetostratigraphy to 17.1 Ma^33^ (Fig. 2). The timing of terrace formation is thus bracketed between the deposition of the youngest early Oligocene Promina Beds (34–28 Ma) and the deposition of the Pag Island Miocene sediments (17.1 Ma) (Figs. 1, 2, 3).

The depth of the up to 150 km deep high velocity P-wave tomography body underneath the central Dinarides^21,23,36^ is in agreement with independent surface S-wave tomography^42^ and with SKS splitting data, which show northward mantle flow at depths of > 150 km^26^. This uniform northward mantle flow, captured by the average fast SKS across the central External Dinarides, shows no switch of the asthenosphere mantle flow direction even in close proximity to the top of the high velocity body (Fig. 1). Within the margin of error, the comparison of the SKS data, the S-wave and P-wave tomography show no evidence of a slab deep enough to disturb the mantle flow pattern in the central Dinarides as observed in the Alps and Apennines^43^. However, the top of the leading edge of this high P-wave velocity body underneath the entire Dinarides (Fig. 1b, supplementary videos) correlates with the pronounced change in crustal^44^ and in the northern to central Dinarides also the lithospheric thickness^45^ . All three of these abrupt lithosphere-scale changes show a first order correlation with the Mediterranean-Black Sea drainage divide across the Dinarides (Figs. 1b, 6).

The four river pairs across the drainage divide reveal up to 380 m of symmetric incision in the catchments on either side (Fig, 1c). Disruption on one side of the drainage divide within a catchment would have led to an asymmetrical incision, which we do not observe. We therefore interpret river incision amounting to 150–380 m as a response to an Oligo-Miocene uplift, responsible also for the mean uplift of 200–300 m of the marine terraces (Fig. 4). Consequently, the area of uplift was not only restricted to the Adriatic coastline, but rather affected the entire Dinarides.

## Discussion

### Neogene (post uplift) shortening in the Dinarides

Although the lack of suitable marker beds younger than the Miocene Dinaric Lake Sediments make it difficult to quantify the exact amount of Neogene shortening in the Dinarides, several studies show a substantial amount of post-uplift Neogene shortening^17,46,47^. For instance, palinspastic reconstructions show a 5° + /− 2° counter clockwise (CCW) rotation of Adria, which is associated with about 100 km of post-20 Ma shortening in the Dinarides^47^. This is supported by the detailed structural analysis from the Internal and southern External Dinarides, which show evidence for post-18 Ma N-S shortening^17,46^. Bennett, et al.^48^ proposed an Eocene to present northeastward subduction of continental lithosphere underneath the Dinarides to explain the substantial decrease of the present-day GPS velocities from the External to the Internal Dinarides.

**Figure 5:**
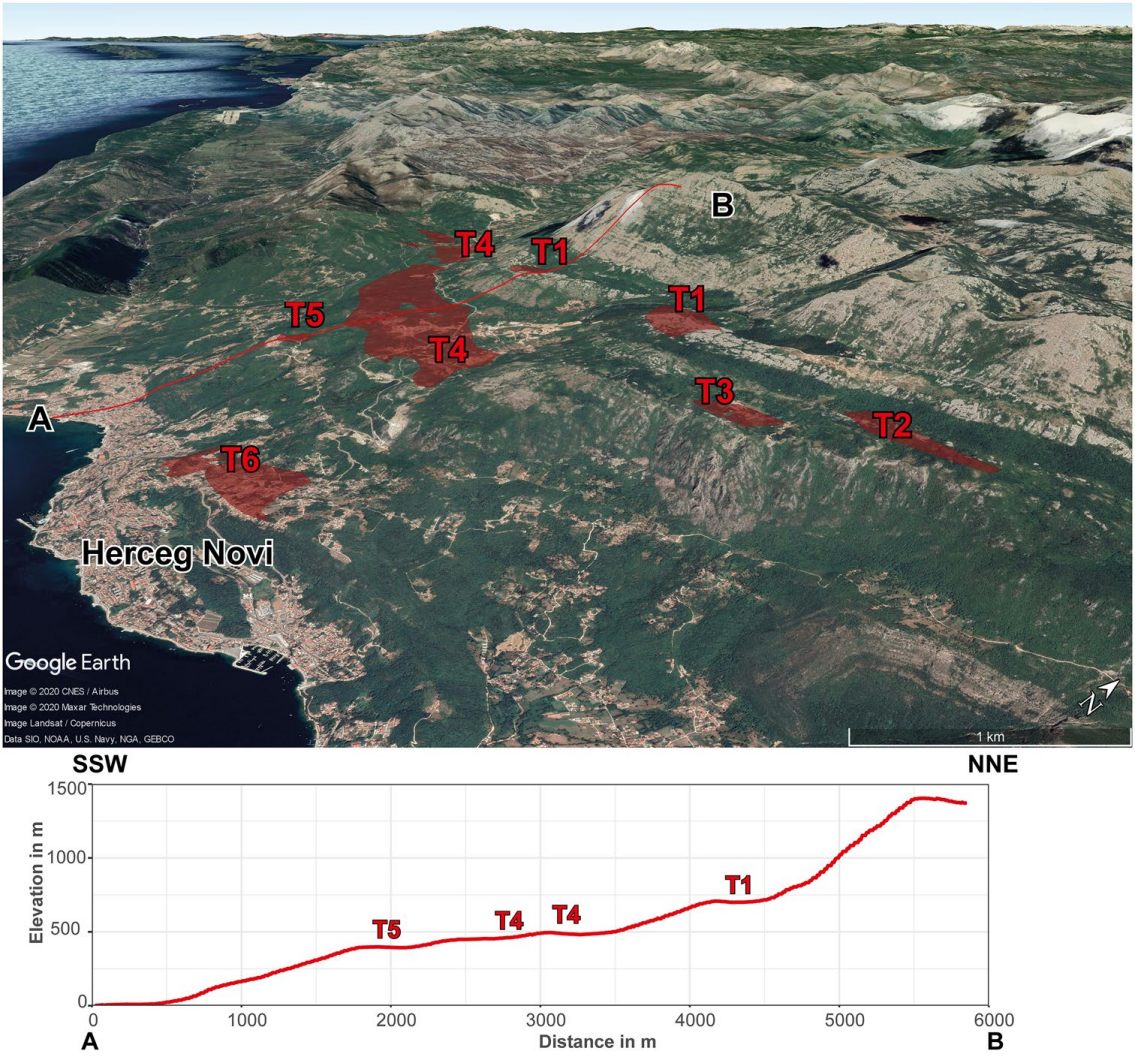
Staircase morphology in the southern External Dinarides. Satellite image draped on top of a DEM (Google Earth) of the southern External Dinarides in northwestern Montenegro. The mapped terraces are marked in red and annotated by their relative age, where T1 marks the oldest and T6 the youngest terrace. The lower panel shows the staircase morphology on a topographic profile A–B with the terraces annotated with their relative age.

In contrast, AMS (anisotropy of magnetic susceptibility) data from the Neogene Dinaric lake sediments show evidence for mild NE-SW shortening, but do not confirm a CCW rotation of the Dinarides since their deposition (18–13 Ma)^32^. Negligible Neogene shortening in the central External Dinarides is also supported by the absence of a Miocene flexural foreland basin (Fig. 1a) and the lack of Miocene low–temperature cooling ages in the central External Dinarides^49^ (Fig. 2). In addition, the pristine preservation of the horizontal 28–17.1 Ma old marine terraces presented here provides evidence for the absence of substantial Neogene shortening in the central External Dinarides. Consequently, the Neogene shortening was most likely accommodated either in the more internal part of the Dinarides or within the even more internal Carpathian-Pannonian system. This is documented in the Dinarides—Alpine—Pannonian basin transition zone in Croatia^50^ and in northerly adjacent Slovenia^51^. In contrast, the frequency of the mapped terraces decreases towards the southern Dinarides (Figs. 1, 4). South of the city of Dubrovnik the terraces are scattered and only locally a staircase morphology is preserved (Fig. 5). This might be related to a Neogene structural overprint due to pronounced Neogene shortening in the southern External Dinarides^17,52,53^, which is in agreement with the presence of the Neogene South Adriatic Basin^54^ (Fig. 1a).

Interestingly, the seismic zoning, derived from recent earthquakes, shows the highest probability of strong ground motion in the northern and southern Dinarides, whereas the central External Dinarides are categorized as areas of lower expected ground motion^55^. This is in line with the higher preservation of the mapped terraces in the central External Dinarides (Fig. 4). However, GPS velocities^48^ and fault plane solution data^56^ prove that the present-day Adria-Europe convergence^48,57^ has to be accommodated partly by shortening within the entire Dinarides fold and thrust belt^48,58^ and in the Dinarides–Pannonian basin transition area^59^.

### Deep-seated processes governing the Oligo-Miocene surface uplift

**Figure 6:**
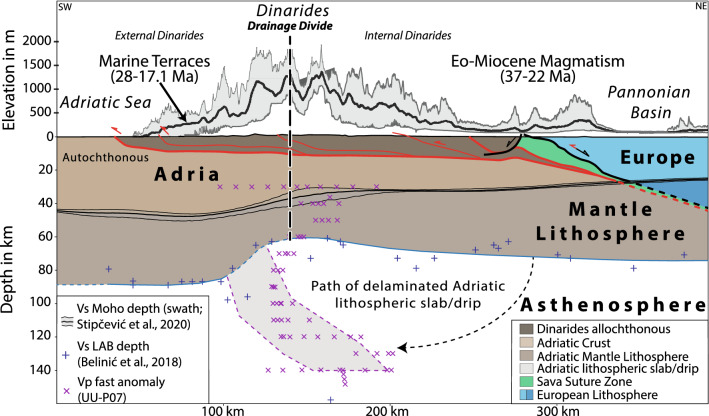
Lithosphere-scale cross-section across the Dinarides fold and thrust belt. Topographic and Moho depth swath profiles (50 km width) along the section trace (location Fig. 1b). Main detachments of mainly Paleogene nappe stacking (red) and younger partly reactivated extensional detachments in black^16^. The LAB depth is projected into the section trace (location: Fig. 1b). The thinnest lithospheric part corresponds to the drainage divide. The fast P-wave tomographic anomaly is interpreted as a remnant of the “peeled-off” Adriatic lithosphere.

**Figure 7:**
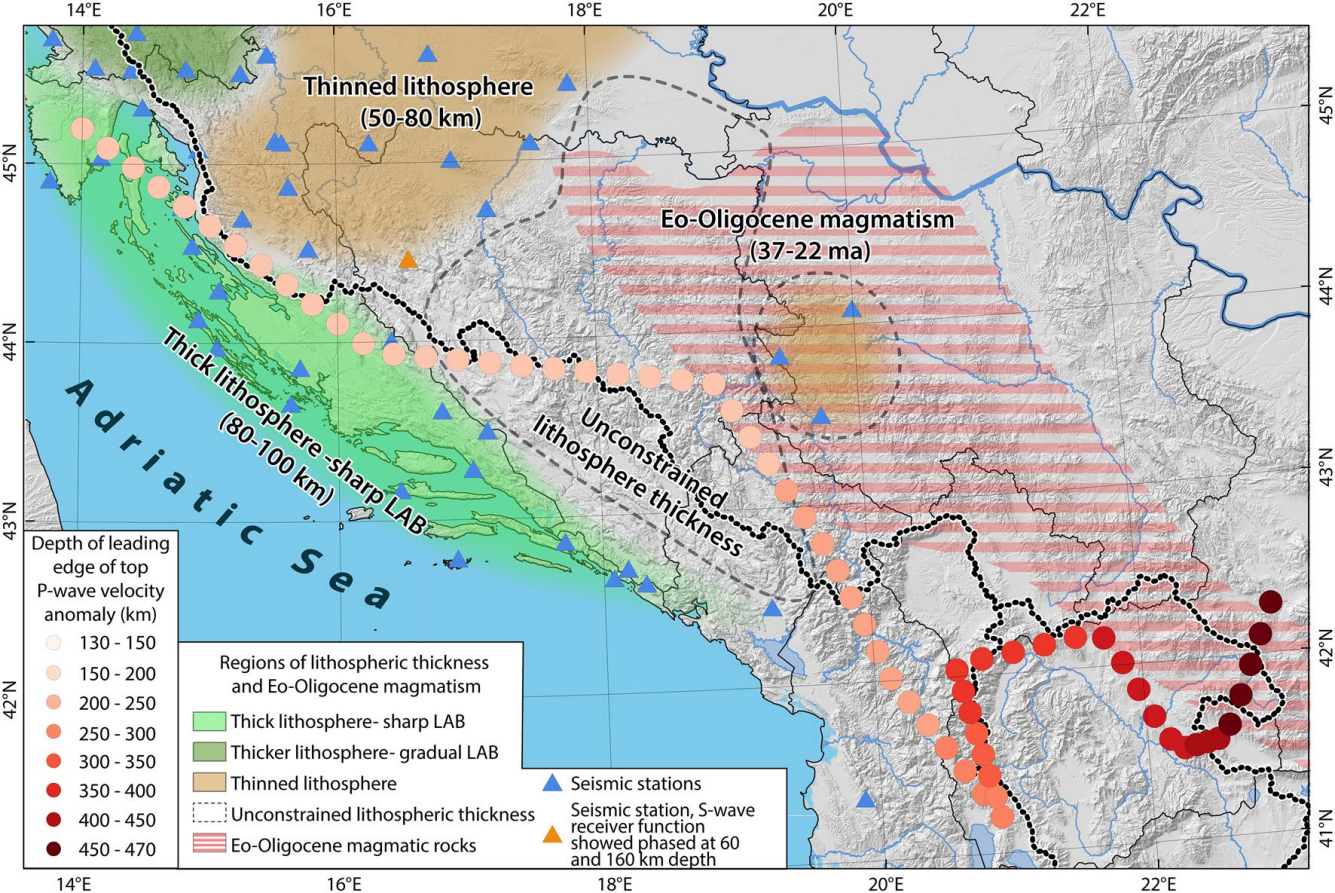
DEM^34^ of the Dinarides showing regions of different lithosphere thicknesses, modified after Belinić, et al.^45^, the domain of Eo-Oligocene magmatic rocks after Handy, et al.^20^, the depth and position of the leading edge of the mapped high velocity P-wave anomaly and the drainage divide. The dark green regions are associated with a thick lithosphere and a gradual LAB (northern Dinarides), the light green regions are related to a thick lithosphere (most External Dinarides) and sharp LAB and the brown regions are related to a thinned lithosphere (Internal Dinarides). The red striped region shows the location of Eo-Oligocene magmatic rocks with mantle affinity.

The extent of the uplifted area and the spatial correlation between the drainage divide and the thinnest part of the Adriatic lithosphere in the central Dinarides^45^ (60 km, Fig. 6) favor a deep-seated process that governs surface uplift^60^. The lithosphere-scale cross-section across the central Dinarides (Fig. 6) shows that the undeformed part of the Adriatic plate is much thicker^45^ (90 km) than the deformed lithosphere^45^ (60–75 km, Fig. 6). This contradicts an expected thickened lithosphere underneath the Dinarides due to Late-Cretaceous to Paleogene nappe stacking and thus pleads for post-collisional removal of the orogenic root. P-wave tomography model UU-P07 reveals an NE-dipping positive anomaly down to 180 km (supplementary videos). Such an orogen-wide lithosphere structure is independently substantiated by the inversion of Rayleigh-wave dispersion data, which shows a positive S-wave velocity anomaly underneath the entire Dinarides^42^. Based on its position and shape we interpret this body as delaminated lithospheric mantle of the Adriatic plate (Fig. 6). We suggest that here, delamination caused inflow of less dense asthenosphere, which led to the elevated Oligo-Miocene Moho and induced surface uplift in the entire External Dinarides. The good correlation of the dipping lithosphere structure with the northeastern margin of thick lithosphere in the northern and north-central part of the External Dinarides further supports delamination in this region (Fig. 7). This correlation becomes less clear in the south-central and southern External Dinarides, where large parts of the Adriatic lithosphere thickness^45^ is undetermined due to the lack of seismic stations (Fig. 7). In this region the lack of geophysical data is supplemented by geochemical data of the Eo-Oligocene magmatism, which shows mantle affinity (Fig. 7). This affinity is interpreted to reflect mantle-crust-interaction^18^ and additionally supports orogen-wide delamination in the Dinarides.

Prelević, et al.^61^ showed that ultra-potassic volcanics from around the Sava Suture zone (Figs. 1a, 4, 7) exhibit a metasomatized mantle affinity that resulted from mantle-crust-interaction. The radiometric ages of these and other igneous rocks in the Internal Dinarides of 37–22 Ma (Figs. 2, 6, 7) clearly post-date Late Cretaceous collision and were previously explained by either late Eocene slab break-off (slab detachment) of the Adriatic slab^62^ or by slab break-off followed by mantle delamination^18^.

Van de Zedde and Wortel^63^ showed that the shallow detachment or break-off, i.e. at depths of 30–50 km, leads to a lateral contrast in buoyancy distribution which may trigger a Bird-type^9^ delamination, leading to a peeled-off Adriatic lithospheric mantle. Andrić, et al.^62^ proved that this model is applicable in the Dinarides, by relating the slab detachment (break-off) to a relamination of the lower crust to explain the foreland propagation of the syn- and post-collisional magmatism in the Internal Dinarides. The results of their best-fit 2D magmatic-thermochemical numerical model show that a progressive lithosphere slab retreat could control the change from Eocene relamination and contraction to Oligocene–Miocene eduction and extension, as observed in the Internal Dinarides and the Sava Zone^62^ (Fig. 2). Other studies, in turn, relate the prominent Oligo-Miocene extension to the contemporaneous subsidence of the northerly adjacent Pannonian Basin^16,64^ (Fig. 2).

Under the assumption that the present-day Adria-Europe convergence was active since late Eocene, Bennett, et al.^48^ calculated a slab length of 138 km underneath the Dinarides to propose an active uninterrupted subduction since late Eocene times and to match the observed up to 180 km deep high velocity P-wave anomaly. This assumption requires a substantial amount of Neogene shortening in the entire Dinarides. However, the preservation of the horizontal staircase morphology presented here rather implies the absence of Neogene crustal shortening during and since the Oligo-Miocene surface uplift for the central External Dinarides.

### Comparison with other studies

Although the preservation of Oligo-Miocene flat erosional surfaces within the Alpine-Himalayan-Belt seems to be exceptional, a Late Miocene (10 Ma) exposure age of the Dachstein Paleosurface was reported from the Northern Calcareous Alps^65^. The formation of the Dachstein paleosurface was dated to Late Eocene to Early Oligocene times, when this surface was buried by the 1.3 km thick Augenstein Formation and experienced a Late Miocene uplift pulse, which led to the exposure of the paleosurface^65^. As with the set of Oligo-Miocene marine terraces reported here, the Dachstein paleosurface is also preserved within a thick carbonate sequence. According to Frisch, et al.^65^ a karstic environment might favor subsurface erosion and formation of caves over surface erosion, which led to the preservation of the Late Miocene exposed geomorphic features in the Northern Calcareous Alps. A similar process of mainly subsurface erosion might have taken place in the Dinarides, where the pristine preservation of the marine terraces in the External Dinarides recorded an Oligo-Miocene delamination event.

Mantle geometries that were explained by delamination patterns were also reported for the east Carpathians, and the Colorado Plateau. In both scenarios delamination occurred without crustal shortening. The uplift of the Colorado Plateau was ascribed to slab removal, which caused thermo-chemical convection leading to presently active delamination, surface uplift, and concomitant incision of the Grand Canyon^11^. Underneath the Colorado Plateau only P-wave tomography confirms the existence of a partly attached foundering continental slab, while it remains undetected by S-wave receiver functions^11^. This is comparable to our case in the Dinarides, where the continental slab is continuously detected by P-wave tomography (Fig. 1), whereas S-wave receiver functions provide evidence for a much deeper reflector in one location only^45^ (Fig. 7).

In the Eastern Carpathians delamination led to surface uplift and foundering of the seismically active, near-vertical lithospheric slab underneath the Vrancea region, which shows eastwards-retreat since collision as a consequence to slab break-off^10^. Numerical models^8^ showed that denser, partly attached oceanic lithosphere may have triggered delamination, which resulted in the observed uplift pattern and subsidence geometries within the Carpathians. The numerical model of Göğüş, et al.^66^, in particular the model run (EXP-6) based on decreased initial mantle lithosphere density, shows that mantle upwelling occurs only within in a limited zone as a response to continental lithosphere removal. This lithosphere removal results in a rather shallow (< 300 km depth) lithospheric slab and a maximum surface uplift of 1,000 m within a 300 km wide zone^66^ perpendicular to the orogen. Different durations of the lithospheric removal of 5.1 Ma, 8.9 Ma, and 10.1 Ma lead to variable slab length but to identical uplift patterns^66^. The slab depth, the amount of associated uplift, and the duration of lithosphere removal fit our observations and proposed time window of lithosphere removal underneath the Dinarides (Fig. 2).

The radiometric ages of igneous and mafic extrusive rocks in the Internal Dinarides, interpreted as proxies for mantle delamination, gradually follow Late Eocene slab detachment and range from 37 to 22 Ma^18,67^. This coincides with the estimated time of surface uplift between 28 and 17.1 Ma (Fig. 2), as inferred from relative age dating of the marine terraces discussed above. Since both are related to mantle delamination, the timing of the uplift can be constrained to 28–22 Ma. The potential causes of such post-collisional continental delamination can be related to either (i) a rather shallow Late Eocene slab break-off, which weakened the lithosphere between the Dinarides and Pannonian-Carpathian system and led to a lateral instable buoyancy distribution; or (ii) by eclogitization due to lithospheric over-thickening. The preservation and the relative dating of the marine terraces in the External Dinarides prove that delamination occurred after an early Oligocene slab break off and was not associated with crustal shortening. Our results indicate that delamination, which may have culminated from a slow incipient to a rapid process, is responsible for the orogen-wide surface uplift of the Dinarides in Oligo-Miocene times.

## Conclusions

One of the most striking conclusions of our study is that marine terraces, formed and uplifted during Oligocene to Miocene (28–22/17.1 Ma) times, are still discernible at present despite a karstic environment, emphasizing the regional importance of this uplift event. The regional surface uplift signal of up to 600 m is restricted to the along-strike extent of the NE edge of a delaminated continental slab. The position of the leading edge of the slab shows a first order correlation with the Mediterranean-Black Sea drainage divide. Symmetric incision across the drainage divide pleads for uniform uplift, with its crest underneath the drainage divide. Relative ages of the terraces coincide with the age of post-collisional magmatism in the Internal Dinarides. The combination of deep geophysical and surface geomorphic data suggests an Oligo-Miocene delamination event following early Oligocene slab detachment leading to Moho- and surface uplift. This delamination is confirmed by a shallow, sub vertical positive P-wave anomaly, where post-collisional mantle delamination began at 28 Ma and terminated 22 Ma ago.

## Supplementary Information

Supplementary Video 1.

Supplementary Video 2.

Supplementary Information 1.

Supplementary Information 2.

Supplementary Legends.

## Data Availability

Tomography model UU-P07 is available at https://www.atlas-of-the-underworld.org.
