# Supplementary material for: Post-collisional mantle delamination in the Dinarides implied from staircases of Oligo-Miocene uplifted marine terraces
Source: Sci Rep. 2021 Jan 29;11:2685. doi: 10.1038/s41598-021-81561-5 (PMC7846848; doi:10.1038/s41598-021-81561-5)
Supplement: Supplementary file 4 — Supplementary Information 2. [file 41598_2021_81561_MOESM4_ESM.zip › Balling_et_al_supplement_A1/Figure_1_3D.html]

Terraces and Slabgeometry Dinarides


×

Layer name

|  |

Clicked coordinates

|  |

Zoom in here

Move here

Orbit around here

Attributes

# Current View URL

# Usage

|  |  |
| --- | --- |
| Mouse | |
| Left button + Move | Orbit |
| Mouse Wheel | Zoom |
| Right button + Move | Pan |
| Keys | |
| Arrow keys | Move Horizontally |
| Shift + Arrow keys | Orbit |
| Ctrl + Arrow keys | Rotate |
| Shift + Ctrl + Up / Down | Zoom In / Out |
| L | Toggle Label Visibility |
| R | Start / Stop Rotate Animation (Orbiting) |
| W | Wireframe Mode |
| Shift + R | Reset Camera Position |
| Shift + S | Save Image |

# About

This page was made with QGIS and Qgis2threejs plugin.
Dependent JavaScript libraries are
three.js,
dat-gui
 and Proj4js
.
