# Supplementary material for: Post-collisional mantle delamination in the Dinarides implied from staircases of Oligo-Miocene uplifted marine terraces
Source: Sci Rep. 2021 Jan 29;11:2685. doi: 10.1038/s41598-021-81561-5 (PMC7846848; doi:10.1038/s41598-021-81561-5)
Supplement: Supplementary file 5 — Supplementary Legends. [file 41598_2021_81561_MOESM5_ESM.docx]

Post-collisional mantle delamination in the Dinarides implied from stair-cases of Oligo-Miocene uplifted marine terraces

Philipp Balling^1*^, Christoph Grützner^1^, Bruno Tomljenović^2^, Wim Spakman^3^ and Kamil Ustaszewski^1^

^1^Institute for Geological Sciences, Friedrich-Schiller-University Jena, Burgweg 11, 07749 Jena, Germany

^2^Faculty of Mining, Geology & Petrol. Engineering, University of Zagreb, Pierottijeva 6, Croatia

^3^Geosciences, Utrecht University, Vening Meineszgebouw A Princetonlaan 8a, 3584 CB Utrecht, Netherlands

^*^Philipp.Balling@uni-jena.de

A1: 3D model of the DEM of the Dinarides and the mapped positive tomographic P-wave anomaly underneath

A2: Dinarides_UU-P07.shallow_sli.mov: Animation of depth slices (0- 400 km) of the P-wave tomographic model (UU-P07) along-strike the Dinarides from Albanian in the south to Slovenia in the north

A3: Dinarides_UU-P07+SPK.mov: Resolutions tests of the P-wave tomography model

A4: Terraces_Dinarides.kml: Flat surfaces extracted from SRTM DEM along the Dinaric coast
